# p30 protein: a critical regulator of HTLV-1 viral latency and host immunity

**DOI:** 10.1186/s12977-019-0501-2

**Published:** 2019-12-18

**Authors:** Ramona Moles, Sarkis Sarkis, Veronica Galli, Maria Omsland, Damian F. J. Purcell, David Yurick, Georges Khoury, Cynthia A. Pise-Masison, Genoveffa Franchini

**Affiliations:** 1 Animal Models and Retroviral Vaccines Section, Vaccine Branch, Center for Cancer Research, National Cancer Institute, National Institutes of Health, Bethesda, MD USA; 2 Department of Microbiology and Immunology, The Peter Doherty Institute for Infection and Immunity, University of Melbourne, Parkville, VIC Australia

**Keywords:** HTLV-1, *orf*-*II*, p30, *Tax*-*orf*-*II*, Adult T-cell leukemia/lymphoma, ATLL, HTLV-1 associated myelopathy/tropical spastic paraparesis, HAM/TSP, Innate immunity, Adaptive immunity

## Abstract

The extraordinarily high prevalence of HTLV-1 subtype C (HTLV-1C) in some isolated indigenous communities in Oceania and the severity of the health conditions associated with the virus impress the great need for basic and translational research to prevent and treat HTLV-1 infection. The genome of the virus’s most common subtype, HTLV-1A, encodes structural, enzymatic, and regulatory proteins that contribute to viral persistence and pathogenesis. Among these is the p30 protein encoded by the doubly spliced *Tax*-*orf II* mRNA, a nuclear/nucleolar protein with both transcriptional and post-transcriptional activity. The p30 protein inhibits the productive replication cycle via nuclear retention of the mRNA that encodes for both the viral transcriptional trans-activator Tax, and the Rex proteins that regulate the transport of incompletely spliced viral mRNA to the cytoplasm. In myeloid cells, p30 inhibits the PU-1 transcription factor that regulates interferon expression and is a critical mediator of innate and adaptive immunity. Furthermore, p30 alters gene expression, cell cycle progression, and DNA damage responses in T-cells, raising the hypothesis that p30 may directly contribute to T cell transformation. By fine-tuning viral expression while also inhibiting host innate responses, p30 is likely essential for viral infection and persistence. This concept is supported by the finding that macaques, a natural host for the closely genetically related simian T-cell leukemia virus 1 (STLV-1), exposed to an HTLV-1 knockout for p30 expression by a single point mutation do not became infected unless reversion and selection of the wild type HTLV-1 genotype occurs. All together, these data suggest that inhibition of p30 may help to curb and eventually eradicate viral infection by exposing infected cells to an effective host immune response.

## Background

In 1977, an unusual cluster of adult T-cell leukemia/lymphoma (ATLL) reported in southwestern Japan suggested the presence of an infectious agent [1]. The existence of the first identified human oncoretrovirus, HTLV-1, was reported by Poietz et al. [2], and Hinuma et al. confirmed the existence of a retrovirus associated with the lymphoproliferative malignancy ATLL the following year [3]. Adult T-cell leukemia is an aggressive and fatal malignancy characterized by a poor prognosis and survival ranging between 5.5 and 13 months for the acute and lymphoma subtypes [4–8]. HTLV-1A, which is also known as the cosmopolitan subtype, is distributed worldwide and is associated not only with ATLL, but also with the neurodegenerative disorder HTLV-1 associated myelopathy/tropical spastic paraparesis (HAM/TSP) [9, 10], uveitis, infectious dermatitis, and polymyositis [11]. An estimated 5–10 million individuals are infected with HTLV-1 worldwide, but only a low percentage of infections progress to HTLV-1 associated diseases, following a long period of latency in most cases [12–14]. In addition, several studies have shown that disease progression is directly associated with viral burden measured as cell-associated viral DNA [15–21].

The 3′ end region of HTLV-1 was initially described as the “pX region” [22], since its role in the regulation of viral replication and persistence was unknown. However, in the past several decades many groups have demonstrated that alternatively spliced mRNAs from the 3′ end of the positive and negative RNA (RNA+/RNA−) strands encode functional proteins [23–26]. One of these viral proteins, p30, is translated from a doubly spliced mRNA containing open reading frame II (*orf*-*II*) [24, 25, 27]. The messenger RNA of HTLV-1A p30 is detectable in ex vivo samples from HTLV-1 infected individuals and in cell lines expressing the virus [24, 28–32], but the evidence for p30 protein expression remains indirect and is based on (1) functional phenotypes induced by overexpression of p30 in vitro, (2) the finding of antibodies against p30 epitopes in few patients’ sera [33, 34], and (3) rare and low T-cell responses in HTLV-1 infected individuals. Of note, a bioinformatics analysis of the p30 amino acid sequence (Additional file 2: Figure S2) reveals that p30 has highly disordered regions from amino acids 75 to 155 and from 197 to 241. Typically, proteins that present intrinsically disordered regions are characterized by a low level of expression [35]. This may explain the difficulty in detecting p30 protein expression in ATLL patients.

The most compelling direct evidence of the essential role of p30 in viral infection in vivo stems from observations using infectious molecular clones of HTLV-1A. ACH and ACH.p30/p13destroy the initiator methionine of p13 and insert a termination codon in the mRNA encoding p30, and when used to infect rabbits, mutations in *orf II* reduced proviral loads and viral persistence [36]. When a viral mutant ACH.30.1 which did not affect p13 expression was studied in rabbits, this mutant had lower proviral loads compared to wild type ACH. In addition, the authors found reversion of ACH30.1 to wild type and evidence of early coexistence of both mutant and wild type virus [37]. In the rhesus macaque model, p30 was found to be essential for HTLV-1A persistence. The virus was able to infect and persist rabbits when p30 expression was specifically targeted by removing the initiation codon of p30 but retained all other viral genes intact (p30KO). In contrast, p30KO was unable to persist in macaques unless the point mutation reverted to wild type [38]. Together, these findings support the hypothesis that the evolution of HTLV-1 resulted in the selection of an essential viral protein barely recognized by the host immune response.

There is evidence, however, that argues against the importance of p30 in HTLV-1 infection. Sequence comparison of HTLV-1A and HTLV-1B (Additional file 1: Figure S1 and Additional file 2: Figure S2) indicates that HTLV-1B lacks the initiating methionine of p30. Unfortunately, there are only a small number of deposited sequences for HTLV-1B [39] and studies of viral mRNAs have not been conducted. It therefore remains possible that an alternatively spliced message could encode a p30 functional homolog in HTLV-1B. Other studies have reported translation termination or the absence of the initiation codon in the *orf II* that encodes p30 in HTLV-1A infected individuals [40, 41]. While this suggests that p30 may not be necessary late in HTLV-1 infection, it does not rule out that p30 is needed early in infection to establish persistence.

Whether absolutely necessary or not, studies have clearly shown that p30 can play a role in viral replication, host immunity, and cellular proliferation. In this review, we summarize the known functions of p30 in the context of HTLV-1 infection and pathogenesis, and identify key research areas for future investigation.

## HTLV-1A *orf II* encodes for p30

### p30 is a nucleolus resident protein

In 1992, two different research groups independently reported the existence of a doubly spliced mRNA, named *Tax*-*orf II*, encoding p30 (also designated as Tof) [25, 27]. The p30 protein is a 243 amino acid peptide, sharing no significant homology with other human proteins. It contains 23% Serine, 12% Arginine (Additional file 1: Figure S1 and Additional file 2: Figure S2), and a theoretical isoelectric point (pH[I]) of 11.71. Additionally, p30 is mainly a nucleolus resident protein [42], and its positive charge might be responsible for its avid interaction with nucleic acids [43].

The nucleolus is a dynamic structure that contains ribosomal RNA and peptides [44]. Nucleolus resident proteins present a specific retention signal, usually characterized by enriched Arginine and Lysine sequences [45]. Kinetic analysis of fused green fluorescent protein (GFP) shows that p30 displays high motility in the nucleus; in contrast, it is static in the nucleolus [42]. The p30 protein localizes to the nucleolar component, and more specifically to the granular compartment (GC). Ghorbel et al. identified the nucleolar retention sequence of p30 (RRCRSR) by demonstrating that mutation of this sequence prevents p30 from localizing to the nucleolus [42] (Additional file 1: Figure S1 and Additional file 2: Figure S2).

Nucleoli are the site of ribosomal biogenesis. Here, pre-ribosomal particles are synthesized in the nucleoplasm and exported to the cytoplasm as mature ribosomal subunits. Interaction between p30 and a component of the 60S ribosomal subunit, L18a, has been reported [42], although its biological relevance remains unclear. Following treatment with Actinomycin D, an RNA transcription inhibitor, measurement of the GFP-p30 recovery kinetics after photobleaching was found to be reduced compared to untreated controls, suggesting that p30 is retained in the nucleolus in a transcription-dependent manner [42]. The importance of the nucleolus in aging, DNA repair, cell cycle, and messenger RNA export has recently been characterized [44, 46–48]. Several viruses encode for nucleolar proteins [49], which regulate replication of the viral genome and affect cellular gene expression. To date, the effect of p30 on these biological functions of the nucleolus has not been investigated.

### p30 transcriptional activity

An important characteristic of p30 is its transcriptional activity. Early experiments demonstrated that p30, fused to the Gal4 DNA-binding domain, activates transcription via a 62–220 amino acid stretch [50]. Additional studies have revealed that CREB-binding protein (CBP)/p300 mediates p30 transcriptional activity. Repression of cellular cAMP responsive element binding (CREB)-responsive element in a dose-dependent manner was noted in p30-expressing cells. Moreover, p30 has been shown to interact with glutathione S-transferase (GST), compromising the kinase-CBP domain known to bind to CREB and Tax. Importantly, p30 is able to disrupt the assembly of the CREB-Tax-p300 complex, which is necessary for HTLV-1 5′ long terminal repeat (LTR) transcriptional activation [50].

HTLV-1 infection deregulates cellular gene expression by directly affecting transcription or through the alteration of post-transcriptional and epigenetic regulators [51–60]. Microarray analyses in T-cells expressing p30 demonstrated changes in gene expression, similar in part to those found in ATLL cells, supporting the hypothesis that the expression of those genes might be involved in cellular transformation. Michael et al. demonstrated the ability of p30 to downregulate genes involved in adhesion, such as integrins and cadherins. Interestingly, they also noted increased expression of genes involved in T-cell activation and apoptosis [61]. A later report by Taylor et al. of p30-dependent transcription identified a different set of 15 human genes that were upregulated and 65 downregulated by p30. Interestingly, analysis of the cytoplasmatic fraction relative to total RNA identified genes that are post-transcriptionally regulated by p30, with 33 genes found to upregulate transcription and 90 genes that downregulate it [62].

The difference in the gene expression patterns found in these studies is likely due to their different experimental conditions. Michael et al. examined long-term p30 expression in Jurkat T-cells by lentiviral infection [61], while Taylor et al. performed microarray analysis with short-term p30 transgene expression via lentiviral transduction in Jurkat T-cells and PBMCs [62]. Notably, long-term expression of p30 was documented to induce cell cycle arrest [63] that by itself alters the expression of several genes [61, 62]. Regardless, both approaches highlight the important role of p30 in altering gene expression. Gene ontology analysis showed that p30 deregulates genes involved in cell cycle progression, apoptosis, signal transduction, cell adhesion, metabolism, DNA repair, and replication. The domain of p30 that influences cellular gene expression, however, has not yet been identified.

### p30 regulates viral latency

**Fig. 1:**
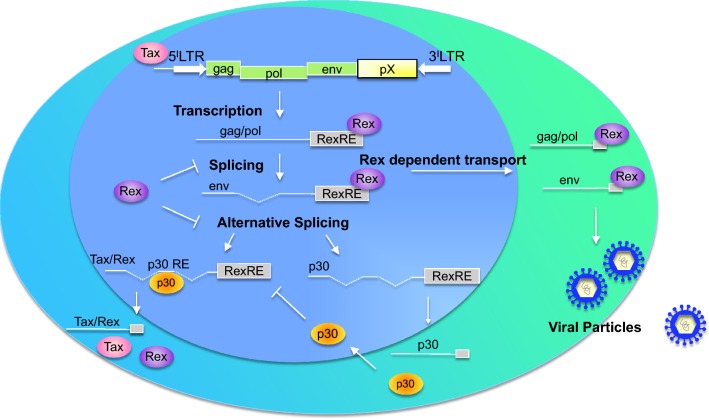
p30 in viral replication. The p30 protein mainly localizes into the nucleolus and represses viral replication by targeting the *Tax/Rex* mRNA. Absent Rex in the early stages of infection, the HTLV-1 viral transcripts are fully spliced, and Tax and Rex are translated. The oncoprotein Tax transactivates the long terminal repeat promoter, amplifying viral expression. Rex exports the unspliced and singly spliced viral mRNA from the nucleus to cytoplasm, leading to the expression of structural proteins and the production of viral particles. In the late stages of infection, p30 directly interacts with Rex, binds the doubly spliced *Tax/Rex* mRNA and decreases both Tax and Rex protein levels, leading to viral latency

HTLV-1 replication leads to the expression of viral regulatory proteins Tax and Rex. Tax activates the transcription of viral genes by interacting with the transcriptional factor CREB to activate the HTLV-1 LTR promoter. Rex is the post-transcriptional regulator of export of the viral mRNAs encoding the structural gag and env proteins and the enzymes (reverse transcriptase, integrase, and protease) necessary to assemble infectious virions (Fig. 1).

The p30 protein is serine-rich, similar to the transcription factors POU-Mi, pit-1, oct1, and oct2 [25]. The post-transcriptional activity of p30 was identified following the observation that overexpression of p30 with an HTLV-1A molecular clone resulted in decreased viral production [64]. Mechanistic analysis revealed that p30 specifically binds the viral *tax/rex* mRNA and facilitates its relative accumulation in the nucleus, thereby suppressing the expression of both the viral trans-activator Tax and of Rex. Therefore, p30 promotes viral latency by reducing Tax and Rex expression [64]. Generation of p30 mutants clearly shows that localization to the nucleolus is not, in fact, necessary for its transcriptional and post-transcriptional activity [42]. The sequence of p30 responsible for the retention of *Tax/Rex* mRNA has not been identified yet, and further studies will be necessary to mechanistically understand the specificity of p30’s interaction with the *tax/rex* mRNA.

In addition to influencing Rex expression, p30 directly interacts with Rex (Fig. 1). The region of p30 between amino acids 131–164 encompasses the Rex binding site (Additional file 1: Figure S1 and Additional file 2: Figure S2) and is not part of p13. While the p13 protein is also encoded by *orf II*, it notably does not bind Rex [43, 65, 66]. Interestingly, the p30/Rex interaction is stronger when p30 is expressed together with an HTLV-1 molecular clone [65], suggesting either a role for viral RNA(s) or that of another viral-induced cellular protein. Mutants of p30 that do not localize to the nucleolus interact with Rex, suggesting that nucleolar localization is not essential for p30/Rex interaction. The domain of Rex involved in p30 binding has been identified by mutation of the six arginines with lysine. The co-expression of the HTLV-1 molecular clone did not rescue the binding of p30 lysine mutant to Rex [65], allowing clear identification of the region of p30 that binds Rex.

Using mass spectrometry, the arginine methyltransferase 5 (PRMT5) was recently identified as a p30 binding partner [56]. Panfil and colleagues investigated the role of PRMT5 in HTLV-1 infection and pathogenesis, showing that this cellular factor mediates cellular transformation and inhibits viral gene expression [67]. PRMT5 levels were found to be elevated in HTLV-1 transformed cells and knockdown of PRMT5 with shRNA or inhibition with a small molecule PRMT5 inhibitor increased HTLV-1 gene expression and decreased cellular proliferation and viability. Since p30 is known to be a negative regulator of HTLV-1 gene expression, the authors investigated the effect of p30 and PRMT5 exogenous expression on the viral LTR. They found that PRMT5 and HTLV-1 p30 had an additive inhibitory effect on HTLV-1 gene expression. However, reduced levels of PRMT5 did not significantly affect the ability of p30 to repress viral transcription, suggesting that the inhibitory role of p30 does not depend on PRMT5 [67].

Together, these studies show that p30 affects the viral life cycle by repressing viral gene expression and promoting the establishment of latency (Fig. 1). This mechanism might permit the virus to avoid recognition by immune cells so persistent infection can be established.

### The p30 protein inhibits the interferon response

Lymphocytes are not the only cell type that HTLV-1 is able to infect. It has been reported that HTLV-1 can also infect monocytes/macrophages and dendritic cells [68–76], but their role in viral pathogenesis is not fully understood. In infected individuals, the majority of viral DNA is found in CD4^+^ and CD8^+^ T-cells. However, a small percentage is observed in all three monocyte subsets defined by CD14 and CD16 expression [77], suggesting that they might contribute to the pathogenesis and/or persistence of the virus.

Different studies have shown that the viral protein p30 modulates the release of cytokines in monocytic cells by affecting the signal of Toll-like receptors (TLRs) [78, 79]. TLRs are mainly activated in response to microbial infection. TLR4 in particular is crucial for dendritic cell maturation and represents an important connection between innate and adaptive immune responses [80–82]. Of note, ATLL patients and HTLV-1C infected Australian Aborigines often present severe immunodeficiency, which correlates with high proviral load and disease progression [83, 84]. This suggests that TLR signaling might be affected in ATLL patients, impairing the innate cells from fully activating the adaptive immune response. Datta et al. demonstrated that the viral protein p30 downregulates TLR4 on the cellular surface, altering the release of pro- and anti-inflammatory cytokines. This effect was found to be mediated by a direct interaction between p30 and the transcription factor PU.1 [78].

**Fig. 2:**
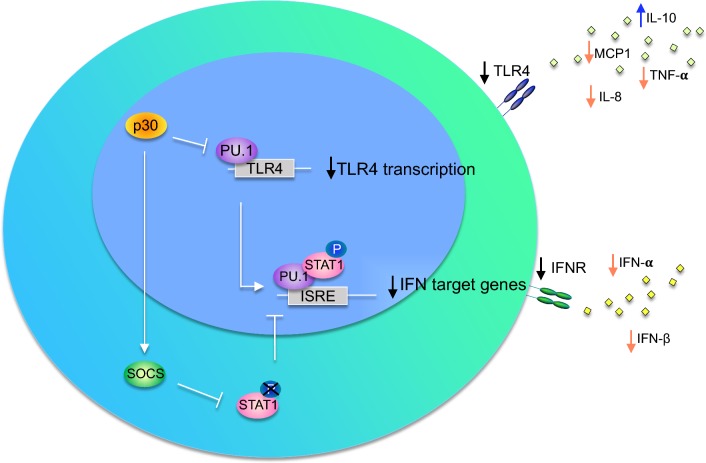
p30 and IFN response. The p30 protein inhibits interferon responsive genes following stimulation by LPS and poly(IC), which respectively activate toll-like receptors TLR4 and TLR3. The p30 protein suppresses the interferon response in a PU.1 dependent manner, leading to reduced STAT1 phosphorylation, probably mediated by the STAT1 negative regulator, SOCS. This molecular event inhibits the release of pro-inflammatory cytokines such MCP1, TNF-α, IL-8, and others, but increases the release of the anti-inflammatory cytokine IL-10 in macrophages

PU.1 is a critical transcription factor that regulates communication among cells of the immune system [85]. Interaction between p30 and PU.1 was first reported in a yeast two-hybrid screen and, subsequentially, in cell lines [78, 79]. Interestingly, p30 interacts with the Ets-domain of PU.1, altering its DNA binding and transcription activity. Since PU.1 regulates its own transcription, expression of p30 results in PU.1 downregulation, thus leading to decreased expression of TLR4 on the cellular surface. This molecular event suppresses the release of the pro-inflammatory cytokines MCP1, TNF-α and IL-8, but increases release of the anti-inflammatory cytokine IL-10 from macrophages following lipopolysaccharide (LPS) stimulation (Fig. 2) [78]. Consistent with this function of p30, high levels of IL-10 in the plasma of ATLL patients and in secretions from most HTLV-1 infected cells is well documented [86, 87].

A more recent study by Fenizia et al. confirmed and expanded on the study by Datta et al. [79] to demonstrate that p30 inhibits interferon responsive genes following stimulation by both LPS and poly(IC), which respectively activate toll-like receptors TLR4 and TLR3 [79]. Using chromatin immunoprecipitation (ChIP) analysis, Fenizia et al. demonstrated that p30 binds PU.1 and decreases its recruitment to the promoters of IFN-responsive genes [78].

Interferons are essential molecules that mediate antiviral innate and adaptive immune responses by affecting cell proliferation, apoptosis, and immune cell activation. Dendritic cells, macrophages, and fibroblasts are the cell types primarily responsible for the production of IFN type I, (α and β). The activation of IFN responses induces upregulation of over 300 genes encoding for immunoregulatory and antiviral proteins [75, 88–91]. It has been shown that primary dendritic cells isolated from infected individuals display reduced IFN secretion, suggesting that HTLV-1 has evolved strategies to escape the interferon response [75]. Consistent with the impairment of IFN expression, suppression of signal transducer and activator of transcription 1 (STAT1) phosphorylation was noted in ex vivo CD4^+^ cells from HTLV-1 infected patients, probably mediated by the STAT1 negative regulator, a suppressor of cytokine signaling (SOCS; Fig. 2) [92, 93]. Interestingly, reduced phosphorylation of TYK2 and STAT2 (members of the IFN cascade) have also been described in infected cells [94–98]. The evolution of the inhibitory effect of p30 on the IFN innate response likely favors viral persistence in immune competent hosts. Overall, these findings support the concept that therapeutic inhibition of p30 functions may improve host recognition of infected cells by increasing viral expression and induce innate and adaptive immune responses to the virus.

### p30 inhibits T-cell proliferation

Recent studies have shown that p30 represses the cellular proliferation of T-cells by delaying their entry into the S phase of the cell cycle and promoting the accumulation of cells in the G2-M phases. P30 has been shown to target multiple G1/S checkpoints in T-cells, thus leading to reduced proliferation [63, 99]. Furthermore, this suggests that p30 may inhibit rapid division of T-cells and thereby suppress the elimination of HTLV-1 infected cells by the host immune system.

**Fig. 3:**
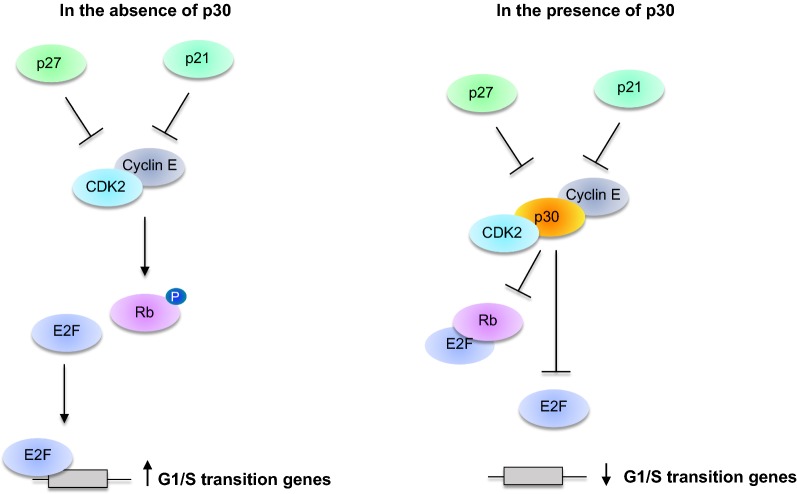
p30 blocks cell cycle progression. In the absence of the p30 viral protein, CDK2 and cyclin E interact and mediate the phosphorylation of Rb. When Rb is phosphorylated, it releases the transcription factor E2F that translocates into the nucleus, and activates the transcription of the G1/S transition genes to promote T-cell proliferation. The viral p30 disrupts the interaction between CDK2 and cyclin E and the ability of this complex to phosphorylate Rb. In this context, E2F is sequestered by Rb and is unable to activate the expression of G1/S transition genes, leading to cell cycle arrest

P30 inhibits cell cycle progression by affecting different regulators. The transition from G1 to the S phase of the cell cycle is regulated by two kinase complexes: CDK4/6-cyclin D and cyclin E-CDK2. During the G1 phase, hypo-phosphorylated Rb sequesters the transcription factor E2F-DP1, blocking the expression of its target genes. Phosphorylation of Rb, mediated by CDK4/6-cyclin D and cyclin E-CDK2, leads to the release of Rb, which activates transcriptional events required for S phase entry (Fig. 3) [100]. Moderate reduction of E2F and cyclin E was observed at the RNA and protein level in p30 expressing cells. However, the molecular mechanism of this p30 dependent repression is still unclear. What is known is that the effect of p30 on cyclin E and CDK2 is associated with decreased phosphorylation of Rb, which may be involved in the late entry into the S phase. Together, these findings suggest that p30 prevents the release of E2F from Rb, reducing the transcriptional activation of E2F target genes involved in the G1/S phase transition (Fig. 3) [63].

The p30 viral protein has also been shown to inhibit cellular proliferation by affecting the expression of the proliferating cell nuclear antigen (PCNA), which is involved in DNA replication and is essential for entry into and the progression of the S phase of the cell cycle [63]. Further, the cyclin-dependent kinase inhibitor, p21 Waf, is upregulated in p30 expressing cells. It is possible that the p30 viral protein (Fig. 5) affects p21 Waf expression by deregulating the transcription factor p53. Ectopic expression of p30 in primary T-cells consistently induces increased expression of the oncosuppressor p53, which is well known to be involved in cell cycle progression and apoptosis. Overexpression of p53 induces cell cycle arrest and accelerates the rate at which apoptosis proceeds [101–103]. Moreover, the p30 viral protein delays progression during the G2/M phases by promoting Checkpoint kinase 1 (CHK-1) phosphorylation, which consequently inhibits expression of the cell cycle regulator, PLK1 [63].

All together, these results show that p30 inhibits the proliferation of infected cells by affecting multiple cell cycle checkpoint regulators. This mechanism is probably important in protecting infected cells from elimination by the immune system.

## P30 promotes the survival of HTLV-1 infected cells

### The p30 protein modulates DNA repair response

**Fig. 4:**
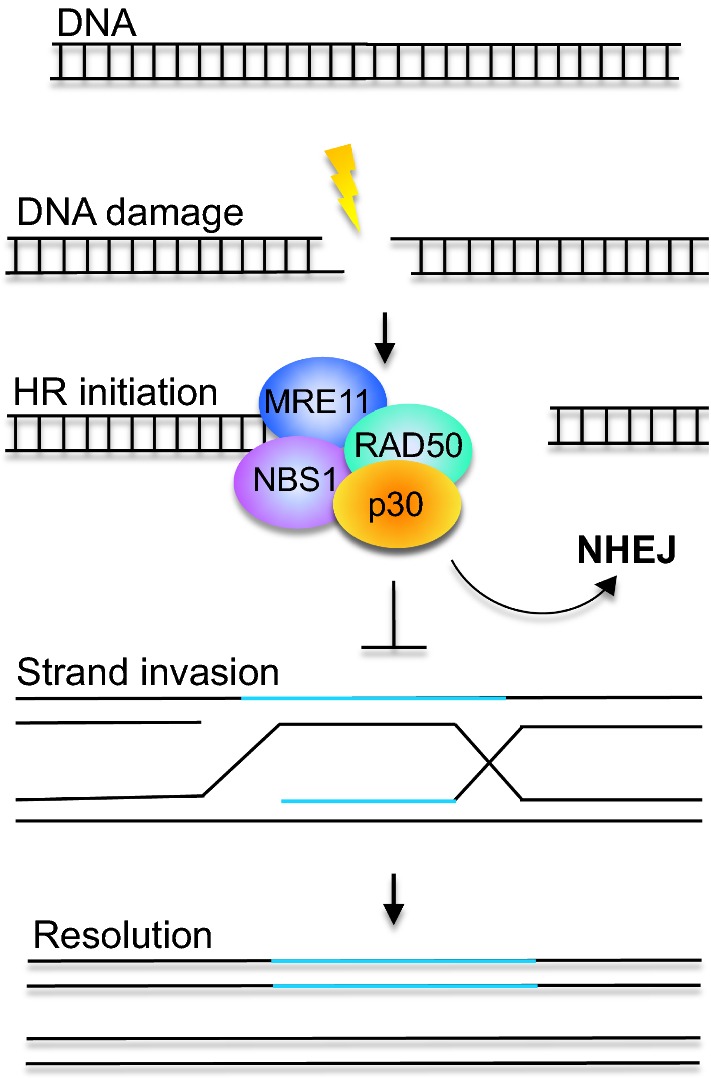
p30 inhibits homologous recombination repair in favor of nonhomologous end-joining repair. DNA damage is an event that is commonly caused by interaction with chemical radicals, produced as a result of cellular metabolism, or by external damaging agents such as ionizing radiations. The broken DNA molecule (black) invades an undamaged homologous molecule (blue) that is used as a template to repair the damage. Repair synthesis is characterized by branch migration, and resolution involving cutting of the junctions between the two molecules (black and blue). The p30 viral protein interacts with the members of the MRN complex, NBS1, and Rad50, essential for the initiation of homologous recombination repair. In the presence of p30, HR repair is impaired, and the DNA double-strand breaks are preferentially repaired through the error-prone NHEJ, which might lead to genetic mutations

The DNA damage response is commonly impaired in human cancers [104–107]. HTLV-1 transformed cells consistently present an increased level of phosphorylated Ataxia telangiectasia mutated (ATM) and H2A histone family member X (H2AX), suggesting the continuous presence of DNA damage [108–110]. DNA double-strand breaks (DDSBs) are a type of DNA damage that typically occur in normal cells after exposure to irradiation and chemicals, leading to the activation of ATM and downstream initiation of the phosphorylation of histone H2AX, a key regulator of DNA damage response. Homologous recombination (HR) repair is an error-free system usually activated during DNA replication that uses homologous template to repair DDSBs. When DNA breaks are generated during the S phase (specifically during lagging DNA strand synthesis or replication fork stalling), HR is activated and the MRN complex (MRE11, RAD50, and NBS1) is recruited at the break sites (Fig. 4). In contrast, nonhomologous end-joining (NHEJ) is a pathway that repairs DDSBs by direct ligation of DNA ends without using a homologous template. NHEJ is an error-prone system because it introduces deletions and is typically activated during the G2 and M phases [111–114]. The viral regulatory protein Tax has an essential role in cellular transformation and has a well-documented effect on DNA repair by inhibiting base excision and homologous recombination repair [115, 116]. Moreover, Tax constitutively activates DNA-PK and attenuates ATM signaling in response to DNA damage [116, 117]. These studies suggest that Tax, by affecting DNA repair, might promote mutagenesis, a crucial event in cellular transformation.

However, the p30 protein has also been shown to affect the DNA repair response favoring the error prone NHEJ, which might promote mutagenesis and oncogenic transformation. Treatment with DNA damaging agents, etoposide, gamma-irradiation, and Bleomycin leads to delocalization of p30 from the nucleolus, suggesting the involvement of p30 in the DNA damage response. By testing p30 mutants, Baydoun et al. demonstrated the existence of a C-terminal motif in the p30 peptide responsible for DDSB-mediated delocalization [118]. In fact, the proline-rich sequence PSTP at the C-terminal of p30 contains an MAPK consensus sequence, with a threonine phosphorylation site. Exposure to MAPK inhibitors abrogates p30 delocalization from the nucleolus in cells exposed to DNA damaging agent. These results were confirmed by using a p30 mutant, T232A, where the Threonine of the PSTP sequence was substituted with an Alanine, indicating an essential role of MAPK in phosphorylating p30 at the PSTP domain upon DNA damage signal [118].

In vitro experiments have shown that p30-expressing cells display nearly 35% deficiency in the homologous recombination repair complex, whereas cells expressing the p30 mutant, T232A, do not. Following exposure to gamma-irradiation, recruitment of the MRN complex, essential for the initiation of HR repair, at the sites of DNA break is impaired in p30-expressing cells. Since the viral protein has been shown to interact with NBS1 and RAD50, two components of the MRN complex, this might explain the deficiency of HR repair in the context of p30 expression (Fig. 4) [118].

Overall, these findings demonstrate that p30 impairs HR repair. However, HTLV-1 infected cells are still able to repair damaged DNA by activating the error-prone system NHEJ. Indeed, a 40% increase in NHEJ activation was observed using an in vivo NHEJ-GFP assay in the presence of p30. These results were confirmed by using an NHEJ-specific inhibitor, Nu7026, that blocks the activity of DNA-dependent protein kinase (DNA-PK), an essential component of the DNA repair system. Following Nu7026 treatment, these HTLV-1^+^ cells accumulate DNA breaks and arrest cells in the S phase. Moreover, immunofluorescence has shown the colocalization of gamma-H2AX, a specific marker of DNA double-strand breaks and DNA-PK, suggesting that the damaged DNA is preferentially repaired by NHEJ when in the presence of p30 [118].

In conclusion, p30 impairs the DNA damage response in HTLV-1 infected cells. Further, treatment with inhibitors that target the DNA repair pathway (PJ45, Olaparib, NSC 19630, and NSC 617145) were found to induce apoptosis not only in HTLV-1 infected cells, but also in ATL-derived cell lines [119, 120], suggesting that DNA repair machinery is impaired in ATL transformed cells and that those drugs might represent a promising therapy for HTLV-1-associated diseases.

### p30 cooperates with the oncogene c-Myc to promote cellular transformation

**Fig. 5:**
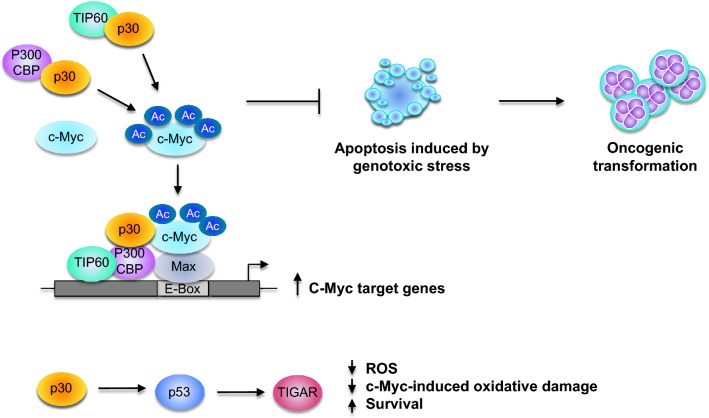
p30 induces c-Myc acetylation that promotes the oncogenic transformation of HTLV-1 infected cells. The viral protein p30 interacts with TIP60 and p300/CBP, inducing acetylation of the oncoprotein c-Myc and leading to the transcription of c-Myc target genes. Moreover, p30 inhibits c-Myc-dependent apoptosis induced by genotoxic stress, which might promote the acquisition of genetic mutations that support oncogenic transformation. In addition, p30 activates the tumor suppressor p53 and induces Tp53-induced glycolysis and apoptosis regulator (TIGAR). Importantly, TIGAR prevents the intracellular accumulation of c-Myc-induced ROS, inhibits oncogene-induced cellular senescence in ATL cells, and promotes cell survival

C-Myc is a well-known proto-oncogene involved in cancer initiation [121] and implicated in the pathogenesis of different types of human tumors [122–124]. In normal conditions, c-Myc activation is restrained to cause tumorigenesis through multiple genetic and epigenetic mechanisms. In many human cancers, c-Myc is overexpressed and associated with proliferation, increased protein biogenesis, activation of angiogenesis, changes in cellular metabolism, and restraint of host immune responses [121]. Overexpression of c-Myc is frequently observed in acute ATL patients as a result of 8q24 chromosomal translocation or *C*-*MYC* locus gene amplification, and it is associated with poor prognosis [125, 126]. The p30 protein has been shown to interact with the MYST-family acetyltransferase TIP60 to promote c-Myc target genes transcription. The p30 amino acid residues 99–154 interact with TIP60, stabilizing the c-Myc-TIP60 on the promoters of c-Myc response genes. C-Myc protein is acetylated on different lysines by p300/CBP, PCAF/GCN5, and TIP60. Acetylation mediated by p300/CBP leads to increased turnover and degradation of the protein (Fig. 5) [126].

P30 is reported to interact with c-Myc and transactivate its E-box enhancer elements within the cyclin D2 promoter. By using shRNA against TIP60 and TIP60 mutants, Romeo et al. demonstrated that in cell lines p30 stabilizes the recruitment of TIP60 to the p30/c-Myc nuclear complex, promoting the transcription of cyclin D2 [127]. The not only affects the progress of the cell cycle by altering the expression of cyclins, but it also induces c-Myc dependent cellular transformation in rodent fibroblasts. In the presence of p30, Lysine to Arginine c-Myc mutants defective for acetylation impaired cellular transformation. Interestingly, both wild type and mutant c-Myc immunoprecipitated with p30, suggesting that the impaired cooperation of c-Myc/p30 in oncogenic transformation depends not on physical interaction, but on the acetylation status of c-Myc. These findings suggested that c-Myc cooperates with p30 to establish cellular transformation in rodent fibroblasts [126].

Using wild type HTLV-1 (HTLV-1_WT_) and p30 defective molecular clones in immortalized primary T-cells, p30 was shown to inhibit apoptosis in the presence of genotoxic stress induced by camptothecin [63]. Interestingly, Romeo et al. demonstrated that p30 inhibits c-Myc dependent apoptosis due to prolongated exposure to BrdU, an inducer of single-strand DNA breaks. Expression of p30 in the presence of the topoisomerase inhibitor leads to multinucleation, possibly due to the activation of c-Myc and p53. Further, expression of p30 induces increased expression of p53 in the presence and in the absence of genotoxic stress (etoposide) and leads to cell cycle arrest (Fig. 5) [127].

Altogether, these findings suggest that p30 might interfere with the C/EBP homology protein (CHOP)-DNA-damaging chemotherapy drugs that are commonly used to treat ATLL by inhibiting apoptosis in the presence of genotoxic stress (Fig. 5). The expression of p30 has the potential to induce somatic mutations that might lead to oncogenic transformation in the early stage of infection and chemotherapy resistance in the advanced phase of the disease.

### p30 induces TP53-induced glycolysis and apoptosis regulator TIGAR

Aberrant activation of specific oncogenes results in metabolic toxicity, which might lead to the cellular transformation necessary for the development of neoplastic disease. The oncogene c-Myc is commonly upregulated in human malignancies [123, 128]. Importantly, p53 is a downstream target of c-Myc. ATLL patients frequently display c-Myc overexpression and the presence of wild type p53 [127].

Romeo et al. showed that p30 augments TP53-induced glycolysis and apoptosis regulator (TIGAR) expression in a p53-dependent manner [127]. The p30 protein was shown to activate p53 by inhibiting its acetylation at K120 and consequentially inducing TIGAR expression. Expression of the viral protein p30 in p53 null-cells is unable to activate TIGAR expression. Elevated TIGAR expression coincides with c-Myc deregulation in primary infected cells isolated from ATLL patients, and, importantly, the induction of TIGAR mediated by p30 prevents c-Myc-induced oxidative damage in HTLV-1 infected cells. More specifically, p30 reduces the oxidative stress, mitochondria damage, and cytotoxicity induced by the HTLV-1 viral proteins Tax and HBZ in a TIGAR-dependent manner. Tax and HBZ increased the cellular level of reactive oxygen species (ROS) and mitochondria membrane depolarization, which is suppressed in the presence of p30. In addition, p30 expression prevents Tax-HBZ induced autophagy and mitophagy [129]. In the context of HTLV-1 molecular clone expression, p30 has the ability to suppress genomic and mitochondria DNA damage.

High TIGAR expression and c-Myc deregulation were observed together in NOD/SCID mice engrafted with the HTLV-1^+^ SLB1 or MET-1 tumor lymphocytes. These animals consistently develop aggressive lymphoid tumors accompanied by enlargement of the liver and spleen. In this model, the high level of TIGAR expression was found to be associated with increased expression of the pro-angiogenic markers VEGF and HIF-1α. Hutchison et al. suggested that TIGAR could promote angiogenesis in HTLV-1 positive tumor cells in the xenograft model, which might play a role in aggressive metastasis and infiltration in secondary tissues of HTLV-1-infected cells [129]. Very little is known about the angiogenesis signal in HTLV-1 pathogenesis, and future studies are needed to better understand the factors involved.

### The p30 protein is essential for HTLV-1 infectivity in the rhesus macaque model

Different laboratories have tried to address the importance of the *orf II* encoding protein p30 in vivo. Ablation of p30 expression does not compromise HTLV-1 replication in primary human cells or its ability to immortalize T-cells in vitro [29, 30]. However, p30 ablation in an HTLV-1 molecular clone did result in abortive infection of primary dendritic cells, suggesting an essential role for the protein in virus replication in myeloid cells [38]. An early study performed with an HTLV-1 molecular clone that had an insertion of 24 bases that truncated p30 and induced a frameshift in the antisense *hbz* open reading frame demonstrated reduced viral infectivity in rabbits. In an additional study in the same species, only two of six animals exposed to the HTLV-1 mutant seroconverted and had reversion to wild type, providing evidence of the coexistence of mutant and wild type viruses in animals that became infected [37]. However, the contribution of the HBZ frameshift could not be ruled out. Later studies were performed with an HTLV-1 molecular clone (HTLV-1_p30KO_) where p30 expression was putatively ablated by a single amino acid mutation in the p30 initiation codon without affecting the expression of the other known HTLV-1 mRNAs [38].

Inoculation of irradiated cells producing HTLV-1_WT_ or the HTLV-1_p30KO_ in rabbits resulted in similar infectivity, suggesting that p30 is not required for persistent infection in this species [38]. DNA isolated from the infected rabbits demonstrated no reversion of the mutation introduced at the initiation codon of the HTLV-1_p30KO_ clone [38]. Rabbits are not naturally infected by HTLV-1, however, and so a similar experiment was performed in rhesus macaques where HTLV-1 is infectious [130]. Interestingly, only one of four macaques inoculated with HTLV-1_p30KO_ fully seroconverted. Two macaques recognized a single viral protein, and one failed to recognize any HTLV-1 antigen. The animals with partial or complete seroconversion were found to be positive for infection by DNA PCR and reversion of HTLV-1_p30KO_ to HTLV-1_WT_ was observed [38]. These findings demonstrate that p30 is essential for the infectivity and persistence of the virus in non-human primates. The results are not unexpected given the profound effect p30 likely exerts on the host immune response. Functional studies on p30 in vitro demonstrate its ability to block IFN responses and T-cell proliferation, which are both essential features of innate and adaptive responses to pathogens. In addition, the ability of p30 to dysregulate the balance between pro- and anti- inflammatory cytokines [78, 79] may create an inflammatory milieu that favors the development of HTLV-1 associated diseases. All together, these data support the importance of p30 in protecting the HTLV-1 infected cells from immune recognition. Unfortunately, very little is known about the expression of p30 during HTLV-1 infection in vivo, and future studies in this animal model are needed to validate the impact of p30 throughout viral infection and disease progression.

## HTLV-1C and HTLV-2 encode for viral proteins with high homology to p30

### HTLV-1C *orf II*

The HTLV-1 C subtype common in central Australia is ancient, but it has recently attracted widespread attention due to its alarmingly high prevalence of nearly 30% infection among the region’s aboriginal population. Mortality at a young age is elevated in HTLV-1C patients, and this virus represents a medical emergency. Molecular studies have identified HTLV-1C as a highly divergent strain of the virus, with the highest divergence found in the 3′end of the viral genome [131–133]. In addition to ATLL and HAM/TSP, individuals infected with HTLV-1C develop lung inflammation, bronchiectasis, and infectious diseases at a high frequency [134–136]. Whether or not there are true differences in the pathogenicity of HTLV-1C and the other HTLV-1 subtypes is unclear, especially given the more than 40,000 years of virus and host co-evolution in some Australian aboriginal communal groups [133]. Similarly, the role of co-morbidities, population genetics, and the diversity observed at the 3′end of the HTLV-1C genome have not been fully investigated.

The p30 amino acid sequence differs between the HTLV-1A and C subtypes. Because p30 functionally affects cell cycle progression, host immune response, and oncogenic transformation, it is possible that the putative HTLV-1C p30 could influence disease outcome. We performed an amino acid comparison analysis of *orf*-*II* in both subtypes using the sequences of 160 individuals infected with HTLV-1A (Additional file 1: Figure S1) [77], 22 Australian patients infected with HTLV-1C [137], and previously published HTLV-1C sequences [131–133, 138]. A consensus was generated among the p30 sequences of the two subtypes (Additional file 2: Figure S2). The p30 of HTLV-1C (p30C) presents amino acid mutations in all the previously identified functional domains: p300 binding site, TRE transcription repression, TIP60 binding domain, Rex binding domain, nucleolus retention sequence (NoRS), and nuclear localization sequence (NLS). However, limited changes were observed in the nuclear localization sequence, suggesting that putative p30C could localize in the nucleus. Interestingly, the NoRS of subtype C has a higher Arginine content than the cosmopolite subtype A, suggesting that p30C might be more static in the nucleolus, affecting its ability to deregulate DNA damage responses. Moreover, mutations at K106, required for p30 transcription repression and T232, necessary for nucleolar and nuclear transport upon DNA damage, are found in different variants of p30C. It is reasonable to speculate that these differences in HTLV-1C p30, especially in DNA repair pathways, may lead to a relatively lower frequency of ATL as observed in those infected in Central Australia [134, 135, 139, 140].

Further studies are needed to fully investigate the biological consequences of the HTLV-1 subtypes. The differences in the p30 of the two HTLV-1 A and C presents one such opportunity to investigate the influence of p30 on viral pathogenesis, host immunity, and viral latency.

### HTLV-2 *orf II* encodes for p28

The *orf II* gene of HTLV-2 encodes for the viral protein p28, shares many characteristics with p30 [141]. Both HTLV-1 p30 and HTLV-2 p28 are important in the regulation of viral replication and persistence, which might affect pathogenic outcome [37, 38, 50, 64, 142]. Both viral products are encoded by a doubly spliced mRNA from the *orf II* and are reported to be unnecessary for infectivity and T-cell transformation in vitro. However, in vivo studies clearly show that both p30 and p28 are needed for the establishment of viral persistence [36–38]. Both proteins have a nuclear and nucleolar localization domain [141, 143] and are negative regulators of viral gene transcription through the retention of Tax/Rex mRNA in the nucleus [64, 141]. However, differences in the functions of these protein have been reported. Unlike p30, p28 is unable to bind cyclin E following transient expression unless it is extremely overexpressed. Cyclin E is involved in cell cycle progression, and it has consistently been shown that p28 does not inhibit the progress of cell transition from the G1 to S phases as p30 does [99]. Further differentiating it from p30, and consistent with the fact that HTLV-2 is not associated with human malignancies, p28 does not affect HR repair [118]. Further study is necessary to address the similarities and differences of these HTLV proteins.

## Conclusions

The HTLV-1 p30 protein has evolved several functions devoted to protecting infected cells from immune recognition. In myeloid cells, p30 favors IL-10 release and inhibits pro-inflammatory cytokines in a PU.1 dependent manner [78, 79]. Its ability to directly compete for DNA binding with the PU.1 transcription factor, a critical regulator of host responses, likely has consequences far beyond interferon responses. PU.1 also regulates the expression of cytokines and chemokines, affecting the communication of immune cells with the microenvironment. It has been speculated that repression of PU.1 or mutations might lead to leukemogenesis and unresponsiveness of leukemic cells to the microenvironment [144]. Thus, the effect of p30 on PU.1 activity may be linked to p30’s role in DNA damage response and repair [85], in addition to its primary function to minimize the innate and adaptive response to HTLV-1. Ultimately, p30 may contribute to the development of leukemia/lymphoma in HTLV-1 infected patients. Further animals studies are needed to determine the consequences of p30 binding to the Tax/Rex mRNA and inhibition of Tax-CBP/p300 complex formation [50, 64], which might reduce viral replication in vivo, and consequently the chance of infected cells to be recognized by the host innate responses. The ability of p30 to inhibit cell cycle progression could hinder the adaptive T-cell immune responses that expand to fight infection. Together, these p30 functions might explain the loss of fitness of the HTLV-1_p30KO_ virus that is unable to persist in the host.

The elevated prevalence of HTLV-1C infection in Australia highlights the importance of basic and translation research to develop effective treatment and prevention strategies. In this review, we have shown the homology between p30 in HTLV-1 A and C (Additional file 2: Figure S2). Point mutations were noted that might compromise the function of p30C or its interaction with other HTLV-1 regulatory proteins, such as Tax and Rex. Studying the biological functions of p30C will allow us to better understand the infectivity, transmission, and pathogenesis of this reemerging infection in Oceania. Moreover, it will be invaluable to identify the precise regions of this viral peptide responsible for specific phenotypes already characterized in the more frequent HTLV-1A subtype.

## Supplementary information

**Additional file 1: Figure S1.** Amino acid sequence analysis of p30 from HTLV-1A infected individuals. Alignment of p30 amino acid sequences of from 160 HTLV-1A patients was used to generate a consensus. Dashes (–) indicate gaps in the amino acid alignment, asterisks (*) represent stop codons, and periods (.) represent similarity. The multi-alignment was performed with the Mega7 program using default parameters.

**Additional file 2: Figure S2.** Amino acid sequence analysis of p30 from HTLV-1A infected individuals. Alignment of p30 amino acid sequences of from 160 HTLV-1A patients was used to generate a consensus. Dashes (–) indicate gaps in the amino acid alignment, asterisks (*) represent stop codons, and periods (.) represent similarity. The multi-alignment was performed with the Mega7 program using default parameters. Amino acid sequence analysis of p30 from HTLV-1A infected individuals. Alignment of p30 amino acid sequences of from 160 HTLV-1A patients was used to generate a consensus. Dashes (–) indicate gaps in the amino acid alignment, asterisks (*) represent stop codons, and periods (.) represent similarity. The multi-alignment was performed with the Mega7 program using default parameters.

## Data Availability

Not applicable.

## Abbreviations

ATL: adult T-cell leukemia
ATLL: adult T-cell leukemia/lymphoma
ATM: ataxia telangiectasia mutated
CBP: CREB binding protein
ChIP: chromatin immunoprecipitation
CHK: checkpoint kinase
CHOP: C/EBP homology protein
CREB: cAMP responsive element binding
DDSB: DNA double-stand break
DNA-PK: DNA-dependent protein kinase
GC: granular compartment
GFP: green fluorescent protein
GST: glutathione S-transferase
H2AX: H2A histone family member X
HAM/TSP: HTLV-1 associated myelopathy/tropical spastic paraparesis
HR: homologous recombination
HTLV: human T-cell leukemia virus
IFN: interferon
KO: knockout
LPS: lipopolysaccharide
LTR: long terminal repeat
NHEJ: nonhomologous end-joining
NLS: nuclear localization sequence
NoRS: nucleolus retention sequence
Op-18: stathmin/oncoprotein 18
*orf*: open reading frame
PCNA: proliferating cell nuclear antigen
pH(I): isoelectric point
PLK: polo-like kinase
PTLV: primate T lymphotropic virus
PRMT5: protein arginine methyltransferase 5
ROS: reactive oxygen species
SOCS: suppressor of cytokine signaling
STAT: signal transducer and activator of transcription
STLV: simian T-cell leukemia virus
TIGAR: TP53-induced glycolysis and apoptosis regulator
TLR: toll-like receptor
*Tof*: *Tax*-*orf II*
TP: tumor protein
