# Supplementary material for: p30 protein: a critical regulator of HTLV-1 viral latency and host immunity
Source: Retrovirology. 2019 Dec 18;16:42. doi: 10.1186/s12977-019-0501-2 (PMC6921414; doi:10.1186/s12977-019-0501-2)
Supplement: Supplementary file 2 — Additional file 2: Figure S2. Amino acid sequence analysis of p30 from HTLV-1A infected individuals. Alignment of p30 amino acid sequences of from 160 HTLV-1A patients was used to generate a consensus. Dashes (–) indicate gaps in the amino acid alignment, asterisks (*) represent stop codons, and periods (.) represent similarity. The multi-alignment was performed with the Mega7 program using default parameters. Amino acid sequence analysis of p30 from HTLV-1A infected individuals. Alignment of p30 amino acid sequences of from 160 HTLV-1A patients was used to generate a consensus. Dashes (–) indicate gaps in the amino acid alignment, asterisks (*) represent stop codons, and periods (.) represent similarity. The multi-alignment was performed with the Mega7 program using default parameters. [file 12977_2019_501_MOESM2_ESM.pdf]

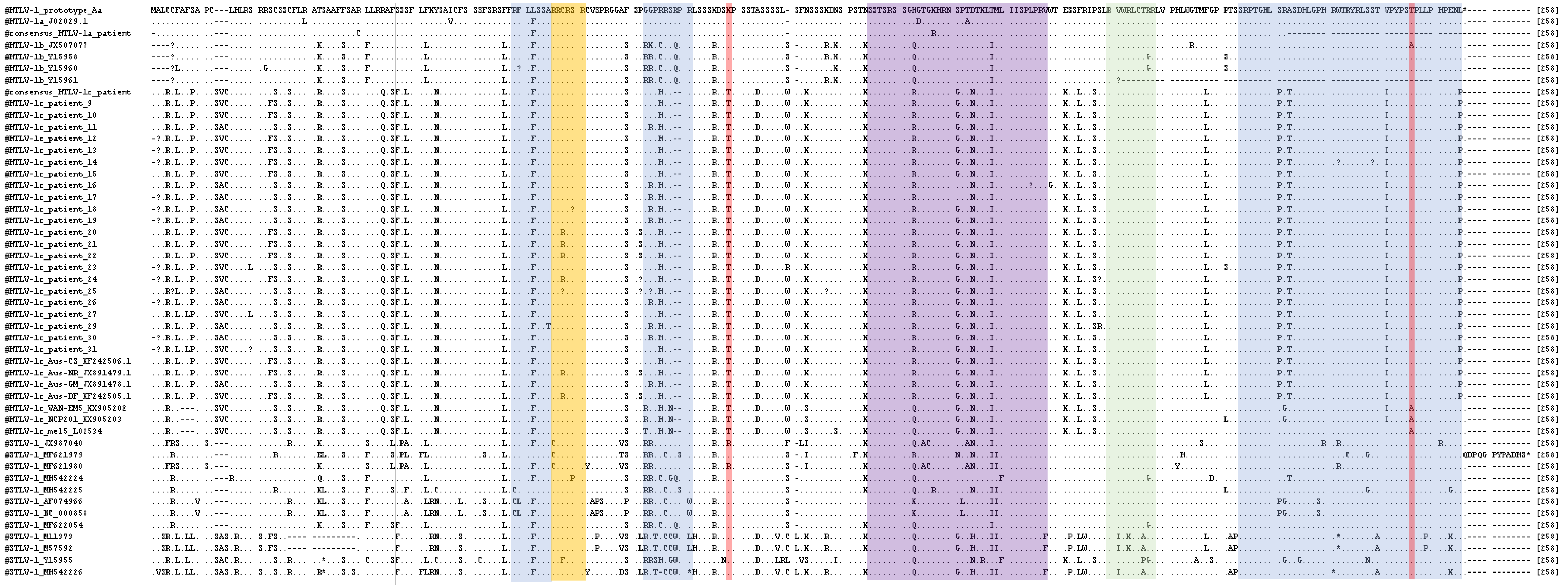
#HTLV-1_prototype_Aa
#HTLV-1a_J02029.1
#consensus_HTLV-1a_patient
#HTLV-1b_JX507077
#HTLV-1b_Y15958
#HTLV-1b_Y15960
#HTLV-1b_Y15961
#consensus_HTLV-1c_patient
#HTLV-1c_patient_9
#HTLV-1c_patient_10
#HTLV-1c_patient_11
#HTLV-1c_patient_12
#HTLV-1c_patient_13
#HTLV-1c_patient_14
#HTLV-1c_patient_15
#HTLV-1c_patient_16
#HTLV-1c_patient_17
#HTLV-1c_patient_18
#HTLV-1c_patient_19
#HTLV-1c_patient_20
#HTLV-1c_patient_21
#HTLV-1c_patient_22
#HTLV-1c_patient_23
#HTLV-1c_patient_24
#HTLV-1c_patient_25
#HTLV-1c_patient_26
#HTLV-1c_patient_27
#HTLV-1c_patient_29
#HTLV-1c_patient_30
#HTLV-1c_patient_31
#HTLV-1c_Aus-CS_KF242506.1
#HTLV-1c_Aus-NR_JX891479.1
#HTLV-1c_Aus-GM_JX891478.1
#HTLV-1c_Aus-DF_KF242505.1
#HTLV-1c_VAN-EM5_KX905202
#HTLV-1c_NCP201_KX905203
#HTLV-1c_mel5_L02534
#STLV-1_JX987040
#STLV-1_MF621979
#STLV-1_MF621980
#STLV-1_MH542224
#STLV-1_MH542225
#STLV-1_AF074966
#STLV-1_NC_000858
#STLV-1_MF622054
#STLV-1_M11373
#STLV-1_M57592
#STLV-1_Y15955
#STLV-1_MH542226
[258]
