# Supplementary material for: p30 protein: a critical regulator of HTLV-1 viral latency and host immunity
Source: Retrovirology. 2019 Dec 18;16:42. doi: 10.1186/s12977-019-0501-2 (PMC6921414; doi:10.1186/s12977-019-0501-2)
Supplement: Supplementary file 1 — Additional file 1: Figure S1. Amino acid sequence analysis of p30 from HTLV-1A infected individuals. Alignment of p30 amino acid sequences of from 160 HTLV-1A patients was used to generate a consensus. Dashes (–) indicate gaps in the amino acid alignment, asterisks (*) represent stop codons, and periods (.) represent similarity. The multi-alignment was performed with the Mega7 program using default parameters. [file 12977_2019_501_MOESM1_ESM.pdf]

```
#consensus_HTLV-1a_patients MALCCFAFSA PCLHLRSRRS CSSCFLRATS AAFFSACLLR RAFSSSFLFK YSAICFSSSF SRSFFRFLFS SARRCRSRCV SPRGGAFSPG GPRRSRPRLS SSKDSKPSST ASSSSLSFNS SSKDNSPSTN SSTSRSSGHG TGRHRNSPTD TKLTMLIISP LPRVWTESSF RIPSLRVWRL CTRRLVPHLW GTMFGPPTSS RPTGHLSR [208]
#1B_AL      .......... .......... ...R...... ......R... .......... .......... .......... .......... .......... .......... .......... .......... .......... .......E.. A.K....... .......... .......... .......... .......... .......... ........ [208]
#1E_AL      .......... .......... ...R...... ......R... .......... .......... .......... .......... .......... .......... .......... .......... .......... .......E.. ..K....... .......... .......... .......... .......... .......... ........ [208]
#1G_AL      .......... .......Q.. ...R...... ......R... .......... .......... .......... .......... .......... .......... .......... .......... .......... .......E.. ..K....... .......... .......... .......... .......... ..V....... ........ [208]
#1I_AL      .......... .......... ...R...... ......R... .......... .......... .......... ....Y..... .......... .......... .......... .......... .......... .......E.. ..K....... .......... .......... .......... .......... .......... ........ [208]
#1L_AL      .......... .......... ...R...... ......R... .......... .......... .......... P......... .......... .......... .......... ...P...... .......... .......E.. ..K....... .......... .......... .......... .......... .......... .......K [208]
#1M_AL      .......... .......... ...RL..... ......R... .......... .......... .......... .......... .......... .......... .......... .......... .......... .......E.. ..K....... .......... .......... .......... .......... .A........ ........ [208]
#1P_AL      .......... .......... ...R...... ......R... ........S. .......... .......... .......... .......... .......... .......... .......... .......... .......E.. ..K....... .......... .......... .......... .......... .......... ........ [208]
#1Q_AL      .......... .......... ...R...... ......R... .......... .......... .......... .......... .......... .......... .......... .......... .......... .......E.. ..K....... .......... .......... .......... .......... .......... .......K [208]
#1R_AL      .........P .......... ...R...... ......R... .......... .......... .......... .......... .......... .......... .......... .......... .......... .......E.. ..K....... .......... .......... .......... .......... .......... ........ [208]
#1S_AL      .......... ..H....... ...R.....P ......R... .......... .......... .......... .......... .......... .......... .......... .P........ .......... .......E.. ..K.....S. .......... .......... .......... .......R.. .......... ........ [208]
#1U_AL      .......... .......... ...R...... ......R... .......... .......... .......... .......... .......... .......... .......... .......I.. .......... .......E.. ..K....... .......... .......... .......... .......... .......... ........ [208]
#2H_AL      .......... .......... ...Y...... .......... .......... .......... .......... .......... .......... .....Q.... .......... .......... .......... .......... ..K....... .......... .......... .......... .......... .......... ........ [208]
#2I_AL      .......... .......... .......... .......... .......... .......... .......P.. .......... .......... .....Q.... .......... .......... .......... .......... ..K....... .......... .......... .......... .......... .......... ........ [208]
#2M_AL      .......... .......... .......... .......... .......... .......... .......... .......... .......... .....Q.... .......... .......... .......... .......... ..K....... .......... .......P.. .......... .......... .......... ........ [208]
#2O_AL      .......... .......... .......... .......... .......... .......... .......... .......... .......... .....Q.Q.. .......... .......... .......... .......... ..K....... .......... .......... .......... .......... .......... ........ [208]
#2P_AL      .......... .......... .......... .......... .......... .......... .......... .......... .......... .....Q.... .......... .......... .......... .....P.... ..K....... .......... .......... .......... .......... .......... ........ [208]
#2S_AL      .......... .......... ......P... .......... .......... .......... .......... .......... .......... .....Q..P. .......... .......... .......... .......... ..K....... .......... P......... .......... .......... .......... ........ [208]
#7C_AL      .......... .......... .......... .......... .......... .......... .......... .......... .......... .......... .F........ .......... .......... .......... ..K....... .......... .......... .......... .......... .......... ........ [208]
#7F_AL      .......... ....V..... .......... .......... .......... .......... .......... .......... .......... .......... .......... .......... .......... .......... ..K....... .......... .......... .......... .......... .......... ........ [208]
#7L_AL      .......S.. .......... .......... .......... .......... .......... .......... .......... .......... .......... .......... .......... .......... .......... ..K....... .......... .......... .......... .......... .......... ........ [208]
#7N_AL      .......... .......... .......... .......... .......... .......... .......... ......P... .......... .......... .......... .......... .......... .......... ..K....... .......... .......... .......... .......... .......... ........ [208]
#7O_AL      .......... .......... .......... .......... .......... .......... .......... .......... .......... .......... .......... .......... .......... .......... ..K....... .R........ .......... .......... .......... .......... ........ [208]
#7Q_AL      .......... .......... .......... .......... .......... .......... .......... .......... .......... .......... .......... .......... P......P.. .......... ..K....... .......... .......... .......... .......... .......... ........ [208]
#9A_AL      .......... .R........ .......... .......... .V........ .......... .......... .......... .......... .......... .......... .......... .......... .......... ..K....... .......... .......... .......... .......... .......... ........ [208]
#9D_AL      ........P. .......... .......... .......... .V........ .......... .......... .......... .......... .......... .......... .......... .......... .......... ..K....... .......... .......... .......... .......... .......... ........ [208]
#9E_AL      .......... .......... .......... V.....R... .......... .......... .......... .......... .......... .......... .......... .......... .......... .......... .......... ...A...... .......... .......... .......... .......... ........ [208]
#9G_AL      .......... .......... .......... .......... .V........ .......... .......... ....R..... .......... .......... .......... .......... .......... .......... ..K....... .......... .......... .......... .......... .......... ........ [208]
#9H_AL      .......... .......... .......... ..S....... .V........ .......... .......... .......... .......... .......... .......... .......... .......... .......... ..K....... .......... .......... .......... .......... .......... ........ [208]
#9K_AL      .......... .......... .......... .......... .V........ .......... .......... .......... .......... .......... .......... .......... .......... .......... ..K....... ........G. .......... .......... .......... .......... ........ [208]
#9P_AL      .......... .......... .......... ..L....... .......... .......... .......... .......... .......... .......... .......... .......... .......... .......... .......... .......... .......... .......R.. .......... .......... ........ [208]
#10A_AL     .......... .......... .......... .......... ......L... .......... .......... .......... .......... .......... .......... .......... .......... .......... .......... .......... .......... .......... .......... .......... ........ [208]
#10F_AL     .......... .......... .......... .......... .......... .......... .......... .......... .......... .......... .......... .......... .......... .F........ .......... .......... .......... .......... .......... .......... ........ [208]
#10I_AL     .......... .......... ......Q... .......... .......... .......... .......... .......... .......... .......... .......... .......... .......... .......... .......... .......... .......... .......... .......... .......... ........ [208]
#10L_AL     .......... .......... .......... .......... .......... .......... .......... .......... .......... .......... .......... .......S.. .......... .......... .......... .......... .......... .......... .......... .......... ........ [208]
#10N_AL     .......... .......... .......... .......... .......... .......... .......... .......... .......... .......... .......... .......... .......... .......... .....D.... .......... .......... .......... .......... .......... ........ [208]
#10O_AL     .......... .......... .....P.... .......... .......... .......... .......... .......... .......... .......... .......... .......... .......... .......... .......... .......... .........S .......... .......... .......... ........ [208]
#10Q_AL     .......... .......... .......... .......... .......... .......... .......... P......... .......... .......... .......... .......... .......... .......... .......... .......... .......... .......... .......... .......... ........ [208]
#10S_AL     .......... .......... .......... .......... .......... .......... .......... .......... .......... .......... .......... .......... .......... .......... .......... .......... ........P. Q......... .......... .......... ........ [208]
#16D_AL     .......... .......... .......... .......... .V.....P.. .......... .......... .......... .......... .......... .......... .......... .......... .......... ..K....... .......... .......... .......... .......... .......... ........ [208]
#16E_AL     .......... .......... .......... .......... .V........ ........P. .......... .......... .......... .......... .......... .......... .......... .......... .......... ........I. .......... .......... .......... .......... ........ [208]
#16F_AL     .......... .......... .......... .......... .V........ .......PP. .......... .......... .......... .......... .......... .......... .......... .......... .......... .......... .......... .......... .......... .......... ........ [208]
#16G_AL     .......... .......... .......... .......... .V........ ........P. .......... .......... .......... .......... .......... .......... .......P.. .......... ..K....... .......... .......... .......... .......... .......... ........ [208]
#16H_AL     .......... .......... .......... .......... .V........ ........P. ...S...... .......... .......... .......... .......... .......... .......... .......... ..K....... .......... .......... .......... .......... .......... ........ [208]
#16I_AL     .......... .......... .......... .......... .......... ...V...... .......... .......... .......... .......... .......... .......... .......... .......... ..K....... .......... .......... .......... ..G....... .......... ........ [208]
#16M_AL     .......... ...Y...... .......... .......... .......... .......... .......... .......... .......... .......... .......... .........P .......... .......... .......... .......... .......... .......... .......... .......... ........ [208]
#16N_AL     .......... .......... .......... .......... .V........ ........P. .......... .......... .......... .......... .......... .......... .......... .......... .......... .......... .......... .......... .......... .......... ........ [208]
#16O_AL     .......... .......... Y......... .......... .......... ........P. .......... .......... .......... .......... .......... .......... .......... .......... ..K....... .......... .......... .......... .......... .......... ........ [208]
#16R_AL     .......... .......... .......... .......... .V......S. ........P. .......... .......... .......... .......... .......... .......... .......... .......... ..K....... .......... .......... .......... .......... .......... ........ [208]
#16S_AL     .......... .......... .......... .......... .......... ........P. .......... .......... .......... .......... .......... .......... .......... .......... ..K....... .......... .......... .T.....R.. ........Y. .........P ........ [208]
#c2A_AL     .......... .......... .......... .......... .......... .......... .......... .......... .......... .....Q.... .......... .......... .......... .......... ..K....... .......... .......... .......... .......... .......... ........ [208]
#d7S_AL     .......... ......P... .......... .......... .......... .......... .......... .......... .......... .......... .......... .......... .......... .......... ..K....... .......... .......... .......... .......... .......... ........ [208]
#e1C_AL     .......... .......... ...R...... ......R... .......... .......... .......... .......... .......... .......... .......... .......... .......... .......E.. ..K....... .......... .......... .......... .......... .......... ........ [208]
#g7I_AL     .......... .......... .......... .......... .......... .......... .......... .......... .......... .......... .......... .......... .......... .......... ..K....... .......... .......... .......... .......... .......... ........ [208]
#h16J_AL    .......... .......... .......... .......... .......... ........P. .......... .......... .......... .......... .......... .......... .......... .......... .......... .......... .......... .......... .......... .......... ........ [208]
#j9R_AL     .......... .......... .......... .......... .V........ .......... .......... .......... .......... .......... .......... .......... .......... .......... ..K....... .......... .......... .......... .......... .......... ........ [208]
#l16B_AL    .......... .......... .......... .......... .V........ ........P. .......... .......... .......... .......... .......... .......... .......... .......... ..K....... .......... .......... .......... .......... .......... ........ [208]
#q9S_AL     .......... .......... .......... ..L....... .......... .......... .......... .......... .......... .......... .......... .......... .......... .......... ..K....... .......... .......... .......... .......... .......... ........ [208]
#u10E_AL    .......... .......... .......... .......... .......... .......... .......... .......... .......... .......... .......... .......... .P........ .......... .......... .......... .......... .......... .......... .......... ........ [208]
#w10H_AL    .......... .......... ....L..... .......... .......... .......... .......... .......... .......... .......... .......... .......... .......... .......... .......... .......... .......... .......... .......... .......... ........ [208]
#x9F_AL     .......... .......... .......... .......... .V........ .......... .......... .......... .......... .......... .......... .......... .......... ......P... ..K....... .......... .......... .......... .......... .......... ........ [208]
#4D_AH      .......... .......... .......... ......R... .......... .......... .......... .......... .......... .......... .......... .......... .......... .......... .....D.... .......... .......... .......... .......... .......... ........ [208]
#4E_AH      .......... .......... .......... ......R... .......... .......... ........L. .......... .......... .......... .......... .......... .......... .......... .......... .......... .......... .......... .......... .......... ........ [208]
#4H_AH      .......... .......... .......... ......R... .......... .......... .......... .......... .......... .......... .......... .......... .......... .......... .......... .......... .......F.. .......... ..G....... .......... ........ [208]
#4I_AH      .......... .......... .......... ......R... .......P.. .......... .......... .......... .......... .......... .......... .......... ..R....... .......... .......... .......... .......... .......... .......... .......... ........ [208]
#4O_AH      .......... .......... .......... ......R... .......... .......... .......... .......... .......... .......... .......... .......... .......... ...G...... .......... .......... .......... .......... .......... .......... ........ [208]
#4X_AH      .......... .......... .......... ......R... ..S....... .......... .......... .......... .......... .......... .......... .......... .......... ....G..... .......... .......... .......... .......C.. .......... .......... ........ [208]
#8ID_AH     .......... .......... ...S...... ......R... .......... ...V...... .......... .......... .......... .......... .......... .......... .......... .........D ..K.....A. .......... .......... .......... .......... .......... ........ [208]
#8IP_AH     .......... .......... .P........ .......... .V........ .......... .......... .......... .......... .......... .......... .......... .......... .......... ..K....... .......... .......... .......... .......L.. .......... ........ [208]
#8IV_AH     .......... .......... .......... .......... .V........ .......... .......... .......... .......... .......... ...G...... .......... .......... .......... ..K....... .......... .......... .......... .......... .......... ........ [208]
#8IIP_AH    .......... .......... .P........ .......... .V........ .......... .......... .......... .......... .......... .......... .......... .......... .......... ..K....... .......... .......... .......... .......... .......... ........ [208]
#12D_AH     .......... .......... .......... ......R... .......... .......... .......... .......... .......... .......... .......... .......... .......... .......... .......... .R........ .......... .M........ .......... .......... ........ [208]
#12F_AH     .......... .......... .......... ......R... .......... .......... ....S..... .......... .......... .......... .......... .......... .......... .......... .......... .......... .......... .......... .......... .......... ........ [208]
#12I_AH     ......T... .......... .......... ......R... .......... .......... .......... .......... .......... .......... .......... .......... .......... .......... .......... .......... .......... .......... .......... .......... ........ [208]
#12T_AH     .......... .......... .......... ......R... .......... .......... .......... .......... .......... .......... ....G..... .......... .......... .......... .......... .......... .......... .......... .......... .......... ........ [208]
#17C_AH     .......... .......... .......... .......... .......... .......... .......... .......... .......... .......... .......... .......... .....G.... .......... ..K....... .......... .......... .......... .......... .......... ........ [208]
#17D_AH     .......... .......... .......... .......... .......... .......... .......... ..C....... .......... .......... .......... .......... .......... .......... ..K....... .......... .......... .......... .......... .......... ........ [208]
#17F_AH     .......... .......... .......... .......... .......... .......... .......... .......... .......... .......... .......L.. .......... .......... .......... ..K....... .......... .......... .......... .......... .......... ........ [208]
#17G_AH     .......... .......... .......... .......... .......... .......... .......... .......... .......... .......... .......... .......... .......... .......... ..K....... .......... .......... P......... .......... .......... ........ [208]
#17H_AH     .......... .......... .......... .......... .......... .......... .......... .......... .......... .......... .......... .......... ........I. .......... ..K....... .......... .......... .......... .......... .......... ........ [208]
#17N_AH     .......... .......... ......Q... .......... .......... ...V...... .......... .......... .......... .......... .......... .......... .......... .......... ..K....... .......... .......... .......... .......... .......... ........ [208]
#29A_AH     .......... .......... .......... ......R... .......... .......... .......... .......... .......... .......... .......... .........A .......... .......... .......... .......... .......... Q......... .......... .......... ........ [208]
#29H_AH     .......... .......... .......... .......... .......... .......... .......... .......... .......... .......... .......... .......... .......... .......... ..K....... .......... .......... .......... ........Y. .......... ........ [208]
#29T_AH     .......... .......... .......... ......R... .......... .......... .......... .......... .......... .......... .......... .......... ........N. .......... .......... .......... .......... .......... .......... .......... ........ [208]
#f12B_AH    .......... .......... .......... ......R... .......... .......... .......... .......... .......... .......... .......... .......... .......... .......... .......... .R........ .......... .......... .......... .......... ........ [208]
#n12J_AH    .......... .......... .P........ ......R... .......... .......... .......... .......... .......... .......... .......... .......... .......... .......... .......... .......... .......... .......... .......... .......... ........ [208]
#z29J_AH    ...R...... .......... .......... ......R... .......... .......... .......... .......... .......... .......... .......... .......... ........N. .......... .......... .......... .......... .......... .......... .......... ........ [208]
#a2_17E_AH  .......... .......... .......... .......... .......... .......... .........P .......... .......... .......... .......... .......... .......... .......... .......... .......... .......... .......... .......... .......... ........ [208]
#a4_8IIF_AH .......... .......... .......... .......... .V........ .......... .......... .......... .......... .......... .......... .......... .......... .......... ..K....... .......... .......... .......... .......... .....L.... ........ [208]
#a5_8IIH_AH .......... .......... .......... V.....R... .......... .......... .......... .......... .......... .......... .......... .......... .......... .......... .......... .......... .......... .......... .......... ...I...... ........ [208]
#a6_8IIJ_AH .......... .......... .......... ......R..H .V........ .......... .......... .......... .......... .......... .......... .......S.. .......... .......... ..K....... .......... .......... W.H....... .......... .......... ........ [208]
#a7_8IIK_AH .......... .......... .......... V.....R... .......... .......... .......... .......... .......... .......... .......... .......... .......... .......... .......... .......N.. .......... .......... .......... .......... ........ [208]
#a8_8IIQ_AH .......... .......... .......... .......... .V........ .......... .......... .......... .......... .......... .......... .......... .......... .......... ..K....... A.A....... .......... .......... .......... .......... ........ [208]
#3N_TL      .....L.... .......... .......... .......... .......... .......... .......... .......... .......... .......... .......... .......... .......... .......... .......... .......... .......... .......... .......... .......... ........ [208]
#3G_TL      .......... .......... .......... .......... .......... .......... .......... .......... .......... .......... .......... .........P .......... .......... .......... .......... .......... .......... .......... .......... ........ [208]
#3H_TL      .......... .......... .......... .......... .......... .......... .......... .......... .......... .........P .......... .......... .......... .......... .......... .......... .......... .......... .......... .......... ........ [208]
#3P_TL      .......... .......... .......... .......... .......... .......... .......... .......... .......... .......... .......... .......... .......... .......... .......... .......... .......... .M........ .......... .......... ........ [208]
#5C_TL      .......... .......... .......... ..L...R... .......... .......... .......... .......... .......... .......... .......... .......... .......... .......... .......... .......... .......... .......... .......... .......... ........ [208]
#5D_TL      .......... .......... .......... ......R... .......... .......... .......... .......... .......F.. .......W.. .......... .......... .......... .......... .......... .......... .......... .......... .......... .......... ........ [208]
#5E_TL      .......... .......... .......... ......R... .......... .......... .......... .......... .......... .......... .......P.. .......... .......... .......... .......... .......... .......... .......... .......... .......... ........ [208]
#5F_TL      .......... .......... .......... ......R... .......... .......... .......... .......... .......... .......... .......... .......... .......... .......... .......... .......... .......... .......... .......... ......A... ........ [208]
#5K_TL      .......... .......... .......... ......R... .......... .......... .......... .......... .......... .......... .......... .......... .......... .......... .......... .......... .......... .......... .......... .........F ........ [208]
#5N_TL      .......... .......... .......... ......R... .......... .......... .......... .......... .......... .......... .......... .......... .......... .......... .......... .......... .....I.... .......... .......... .......... ........ [208]
#5O_TL      .......... .......... .......... ......R... .........E .......... .......... .......... .......... .......... .......... .......... .......... .......... .......... .......... .......... .......... .......... .......... ........ [208]
#5Q_TL      .....S.... .......... .......... ......R... .......... .......... .......... .......... .......... .......... .......... .......... .......... .......... .......... ......T... .......... ......G... .......... .......... ........ [208]
#5R_TL      .......... .......... .......... ......R... .......... ....R..... .......... .......... .......... .......... .......... .......... .......... .......... .......... .......... .......... .......... .......... .......... ........ [208]
#5S_TL      .......L.. ...R...... .......... ......R... .......... .......... .......... .......... .......... .......... .......... .......... .......... .......... .......... .......... .......... .......... .......... .......... ........ [208]
#5V_TL      .......... .......... .......... ......R... .......... .......... .......... .......... .......... .......... .......... .......... .......... .......... .........G .......... .......... .......... .......... .......... ........ [208]
#5W_TL      .......... .......... .......... ......R... .......... .......... .......... .V........ .......... .......... .......... .......... .......... .......... .......... .......... .........L .......... .......... .......... ........ [208]
#11J_TL     .......L.. .......... .......... ......R... .......... .......... .......... .......... .......... .......... .......... .......... .......... .......... ..K....... .......... ..K....... W......... .......... .......... ........ [208]
#15I_TL     .......... .......... .......... ...L...... .......... .......... .......... .......... .......... .......... .......... .......... .......... .......... .......... .......... .......... .......... .......... .......... ........ [208]
#15L_TL     .......... .......... ...R...... .......... .......... .......... .......... .......... .......... .......... .......... .......... .......... .......... .......... .......... .......... .......... .......... .......... ........ [208]
#15Q_TL     .......... .......... .......... .......... .......... .......... .......... .......... .......... .......... .......... .......L.. .......... .......... .......... .......... .......... .......... .......... .......... ........ [208]
#18A_TL     .......... .......... .......... ....P.R... .......... .......... .......... .......... .......... .......... .......... .......... .......... .P........ .......... .......... .......... .......... .......... .......... ........ [208]
#18B_TL     .......... .......... .......... ......R... .......... .......... .......... .......... .......... .......... .......... .......... .......... .......... .......... .......... .......... .......... .......... .........A ........ [208]
#18H_TL     .......... .......... .......... ......R... .......... .....S.... .......... .......... .......... .......... .......... .......... .......... .......... .......... .......... .......... .......... .......... .......... ........ [208]
#18K_TL     .......... .......... .......... ......R... .......... .......... .........P .......... .......... .......... .......... .......... .......... .......... .......... .......... .......... .......... .......... .......... ........ [208]
#18O_TL     .......... .......... .......... ......R... .......... .......... .......... .......... .......... .......... .......... .......L.. .......... .......... .......... .......... .......... .......... .......... .......... ........ [208]
#18T_TL     .......... .......... .......... ......R... .......... .......... .......... .......... .......... .......... .......... .......... .......... .......... .......... .......... .......... .......... .A........ .......... ........ [208]
#25M_TL     .......... .......... .......... ......R... .......... .......... .......... .......... .......... .......... .......... .......... .......... .......... ..K....... .......... .......... .......... .......... .......... ........ [208]
#25N_TL     .......... .......... .......... .......... .......... .......... .......... .......... .......... .......... P......... .......... .......... .......... .......... .......... ..K....... .......... .......... .......... ........ [208]
#25R_TL     .......... .......... .......... ......R... .......... .......... .......... .......... .......... .......... .......... .......... .......... .......... ..K....... .......... .......... .......... .......... .......... .......K [208]
#27C_TL     .......... .......... .......... .......... .......... .......... P......P.. .......... .......... .......... .......... .......... .......... .......... .......... .......... .......... .......... .......... .......... ........ [208]
#27D_TL     .......... .......... .......... .......... .......... .......... .......... .......... .......... ..H....... .......... .......... .......... .......... .......... .......... .......... .......... .......... .......... ........ [208]
#27E_TL     .......... .......... .......... .......... .......... .......... .......... L......... .......... .......... .......... .P........ .......... .......... .......... .......... .......... .......... .......... .......... ........ [208]
#27G_TL     .......... .......... Y......... .......... .......... .......... .......... .......... .......... .......... .......... .......... .......... .......... .......... .......... .......... .......... .......... .......... ........ [208]
#27L_TL     .......... .......... .......... .......... .......... .......... .......... .......... .......... .......... .......... .......... ....D..... .......... .......... .......... .......... .......... .......... .......... ........ [208]
#27T_TL     .......... .......... .......... .......... ......S... .......... .......... .......... .......... .......... .......... .......... .......... .......... .......... .R........ .......... .......... .......... .......... ........ [208]
#a11E_TL    .......... .......... .......... ......R... .......... .......... .......... .......... .......... .......... .......... .......... .......... .......... ..K....... .......V.. ..K....... W......... .......... .......... ........ [208]
#b11I_TL    .......... .......... .......... ......R... .......... .......... .......... .......... .......... .......... .......... .......... .......... .......... ..K....... .......... ..K....... W......... .......... .......... ........ [208]
#i3J_TL     .......... .......... .......... .......... .......... .......... .......... .......... .......... .......... .......... .......... .......... .......... .......... .......... .......... .......... .......... .......... ........ [208]
#m25A_TL    .......... .......... .......... .......... .......... .......... .......... .......... .......... .......... .......... .......... .......... .......... .......... .......... ..K....... .......... .......... .......... ........ [208]
#o18F_TL    .......... .......... .......... ......R... .......... .......... .......... .......... .......... .......... .......... .......S.. .......... .......... .......... .......... .......... .......... .......... .......... ........ [208]
#r15T_TL    .......... .......... .......... .......... .......... .......... .......... .......... .......... .......... .......P.. .......... .......... .......... .......... .......... ..T....... .......... .......... .......... ........ [208]
#s3M_TL     .......... .......... .......... .......... .......... .......... .......... .......... .......... .......... .......... .......... .......... .......... .......... .......... .......... .......... .......... .A........ ........ [208]
#s3M_TL     .......... .......... .......... .......... .......... .......... .......... .......... .......... .......... .......... .......... .......... .......... .......... .......... .......... .......... .......... .A........ ........ [208]
#t3S_TL     .......... .......... .......... .......... .......... .......... .......... .......... .......... .......... .......... .......... .......... .....F.... .......L.. .......... .......... .......... .......Y.. .......... ........ [208]
#v15C_TL    .......... .......... .......... .......... .......... .......... .......... .......... .......... .......... .......... .......... .......... .......... .......... .......... .......... .......... .....A.... .......... ........ [208]
#y18E_TL    .......... .......... .......... ......R... .......... .......... .......... .......... .......... .......... .......... .........P .......... .......... .......... .......... .......... .......... .......... .......... ........ [208]
#a9_5H_TL   .......... .......... ....L..... ......R... .......... .......... .......... .......... .......... .......... .......... .......... .......... .......... .......... .......... .......... .......... .......... .......... ........ [208]
#a10_15H_TL .......... .......... .......... .......... ........S. .......... .......... .......... .......... .......... .......... .......... .......... .......... .......... .......... .......... .......... .......... .......... ........ [208]
#6I_TH      .......... .......... .......... .......... .V........ .......... .......... .......... .......... .......... .......... .......V.. .......... .......... ..K....... .......... .......... .......... .......... .......... ........ [208]
#6L_TH      ...R...... .......... .......... ......R... .......... .......... .......... .......... .......... .......... .......... .......... .......... .......... .......... .......... .......... .......... .......... .......... ........ [208]
#6O_TH      .......... .......... ...R...... V..S..R... .......... .......... .......... .......... .......... .......... .......... .......... .......... .......... .......... .......... .......... .......... .......... .......... ........ [208]
#6Q_TH      .......... .......... .......... V.....R... .......... ....R..... .......... .......... .......... .......... .......... .......... .......... .......... .......... .......... .......... .......... .......... .......... ........ [208]
#6R_TH      .......... .......... .......... V.....R... .......... .......... .......... .......... .......... .......... ..E....... .......... .......... .......... .......... .......... .......... .......... .......... .......... ........ [208]
#6T_TH      .......... .......... .......... V.....R... .......... ...V...... .......... .......... .......... .......... .......... .......... .......... .......... .......... .......... .......... .......... .......... .......... ........ [208]
#6U_TH      .......... .......... .......... V.....R... .......... ......P... .......... .......... .......... .......... .......... .......... .......... .......... .......... .......... .......... .......... .......... .......... ........ [208]
#6W_TH      .......... .......... .......... V.....R... .......... ........P. .......... .......... .......... .......... .......... .......... .......... .......... .......... .......... .......... .......... .......... .......... ........ [208]
#6X_TH      .......... .......... .......... V...P.R... .......... .......... .......... .......... .......... .......... .......... .......... .......... .......... ..K....... .......... ..K....... .......... .......... .......... ........ [208]
#13C_TH     .......... .......... .......... .......... .......... C......... .......... .......... .......... .......... .......... .......... .......... .......... .......... .......... .......... .......... .......... .......... ........ [208]
#13O_TH     .......... .......... ..P....... .......... .......... .......... .......... .......... .......... .......... .......... .......... .......... .......... .......... .......... .......... .......... .......... .......... ........ [208]
#13S_TH     .......... .......... .......... ..L....... .......... .......... .......... .......... .......... .......... .......... .......... .......... .......... .......... .......... .......... .......... .......... .......... ........ [208]
#13T_TH     .......... .......... .......... .......... .......... .......... ....S..... .......... .......... .......... .......... ....F..... .......... .......... .......... .......... .......... .......... .......... .......... ........ [208]
#14E_TH     .......... .......... .......... .......... .......... .......... .......... .......... .......... .......... .......... .......... ..R....... .......... .......... .......... .......... .......... .......... .......... ........ [208]
#14L_TH     .......... .......... .......... .......... .......... .......... .......... .......... .......... .......... ........R. .......... .......... .......... .........N .......... .......... Q......... .......... .......... ........ [208]
#14N_TH     .......... .......... .....P.... .......... .......... .......... .......... .......... .......... .......... .......... .......... .......... .......... .......... .......... .......... .......... .......... .........T ........ [208]
#14O_TH     .......... .......... .......... ..S....... .......... .......... .......... .......... .......... .......... .......... .......... .......... .......... .......... .......... .......... .......... .......... .......... ........ [208]
#14P_TH     .......... .......... .......... .......... .......... .......... .......... .......... .......... .......... .......... .......... .......... .......... .......... .......... .......... Q......... .......... .......... ........ [208]
#14R_TH     .V........ .......... .......... .......... .......... .......... .......... .......... .......... .......... ........R. .......... .......... .......... .........N .......... .......... Q......... .......... .......... ........ [208]
#14S_TH     .......... .......... .....P.... .......... .......... .......... .......... .......... .......... .......... .......... .......... .......... .......... .......... .......... .......... .......... .......... .......... ........ [208]
#19C_TH     .......... .......... .......... .......... .......... .......... .......... .......... .......... .......... ........G. .......... .......... .......... .......... .......... .......... .......... .......... .......... ........ [208]
#19F_TH     .......... .......... .......... ......R... .......... .......... P......... .......... .......... .......... .......... .......... .......... .......... .......... .......... .......... .......... .......... .......... ........ [208]
#19G_TH     .......... .......... .......... ......R... .......... .......... .......... .......... .......... .......... .......... .......... P......... .......... .......... .......... .......... Q......... .......... .......... ........ [208]
#19I_TH     .......... .......... .......... ......R... .......... .......... .......... .......... .......... .......... .......... .......... .......... .......... .......... .......... ..W....... .......... .......... .......... ........ [208]
#19J_TH     .......... .......... .......... ......R... .......... .......... .......... .......... .......... .......... .......... .......... .......... .......... .......... .......... .......... .......... .I........ .......... ........ [208]
#19K_TH     .......... .......... .......... .......... .......... .......... .......... .......... .......... .......... .......... .......... .....N.... .......... .......... .......... .......... .......... .......... .......... ........ [208]
#20P_TH     .......... .......... .......... ......RP.. .......... .......... .......... .......... .......... .......... .......... .......... .......... ...N...... .......... .......... .......... .......... .......... .......... ........ [208]
#k6E_TH     .......... .......... .......... V.....R... .......... .......... .......... .......... .......... .......... .......... .......... .......... .......... .......... .......... .......... .......... .......... .......... ........ [208]
#p19B_TH    .......... .......... .......... ......R... .......... .......... .......... .......... .......... .......... .......... .......... .......... .......... .......... .......... .......... .......... .......... .......... ........ [208]
#a1_20R_TH  .......... .......... .......... .......... .......... .......... .......... .......... .......... .......... .......... .......... .......... .......... .......... ........R. ......K... .......... .......... .......... ........ [208]
#a3_14C_TH  .......... .......... .......... V.....R... .......... .......... .......... .......... .......... .......... ...N...... .......... .......... .......... .......... ...A...... .......... .......... .......... .......... ........ [208]
```
